# Crystal structure of the BREX phage defence protein BrxA

**DOI:** 10.1016/j.crstbi.2022.06.001

**Published:** 2022-06-08

**Authors:** Izaak N. Beck, David M. Picton, Tim R. Blower

**Affiliations:** Department of Biosciences, Durham University, Stockton Road, Durham, DH1 3LE, UK

## Abstract

Bacteria are constantly challenged by bacteriophage (phage) infection and have developed multitudinous and varied resistance mechanisms. Bacteriophage Exclusion (BREX) systems protect from phage infection by generating methylation patterns at non-palindromic 6 bp sites in host bacterial DNA, to distinguish and block replication of non-self DNA. Type 1 BREX systems are comprised of six conserved core genes. Here, we present the first reported structure of a BREX core protein, BrxA from the phage defence island of *Escherichia fergusonii* ATCC 35469 plasmid pEFER, solved to 2.09 ​Å. BrxA is a monomeric protein in solution, with an all α-helical globular fold. Conservation of surface charges and structural homology modelling against known phage defence systems highlighted that BrxA contains two helix-turn-helix motifs, juxtaposed by 180°, positioned to bind opposite sides of a DNA major groove. BrxA was subsequently shown to bind dsDNA. This new understanding of BrxA structure, and first indication of BrxA biological activity, suggests a conserved mode of DNA-recognition has become widespread and implemented by diverse phage defence systems.

## Highlights

• The crystal structure of BrxA from multi-drug resistant plasmid pEFER of *Escherichia fergusonii* has been solved to 2.09 ​Å.
• BrxA is the first reported structure for a conserved core protein from the widespread BREX phage defence systems.
• BrxA contains two HTH motifs, analogous to DNA-binding domains of diverse phage defence systems, and is shown to bind dsDNA.

## Graphical abstract

**Figure:**
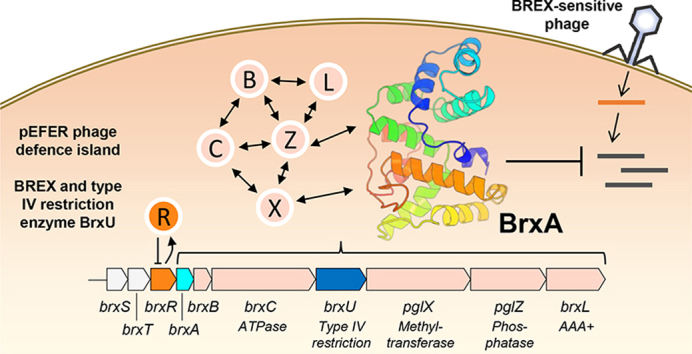

## 1 Introduction

Bacteria must defend themselves from the constant threat of invasion by bacteriophages (phages) and other mobile genetic elements (MGEs). This three-way interaction has driven the evolution of plentiful and diverse modes of protection (Hampton et al., 2020). This includes the long-established restriction-modification (Tock and Dryden, 2005), abortive infection (Blower et al., 2009; Fineran et al., 2009) and CRISPR-*cas* (Barrangou et al., 2007) systems. Recent analyses have identified many new phage defence systems through “guilt-by association” inference of function (Doron et al., 2018), and these diverse systems are often found clustered together into “defence islands” (Makarova et al., 2011).

Bacteriophage Exclusion (BREX) systems (Goldfarb et al., 2015), were previously identified through association of genes with a putative alkaline phosphatase gene, *pglZ*, from Phage Growth Limitation systems (Hoskisson et al., 2015). BREX systems were divided into six sub-types based on associated gene combinations (Goldfarb et al., 2015). The host distribution of BREX systems has been impacted by substantial horizontal gene transfer, although type 1 systems are enriched in *Deltaproteobacteria*, type 2 systems are solely in *Actinobacteria* and type 5 systems are exclusively found in *Halobacteria* archaea (Goldfarb et al., 2015). Type 1 contains six conserved core genes, *brxA*, *brxB*, *brxC*, *pglX*, *pglZ* and *brxL*. Whilst the mechanism of BREX phage defence is currently not understood, it is known that type 1 BREX methyltransferases (PglX) hemi-methylate non-palindromic 6 bp sequences on the N6 adenine nitrogen at the fifth position of the motif (Goldfarb et al., 2015; Gordeeva et al., 2019; Picton et al., 2021). This marks host DNA, leaving incoming non-methylated DNA susceptible to BREX attack.

**Fig. 1:**
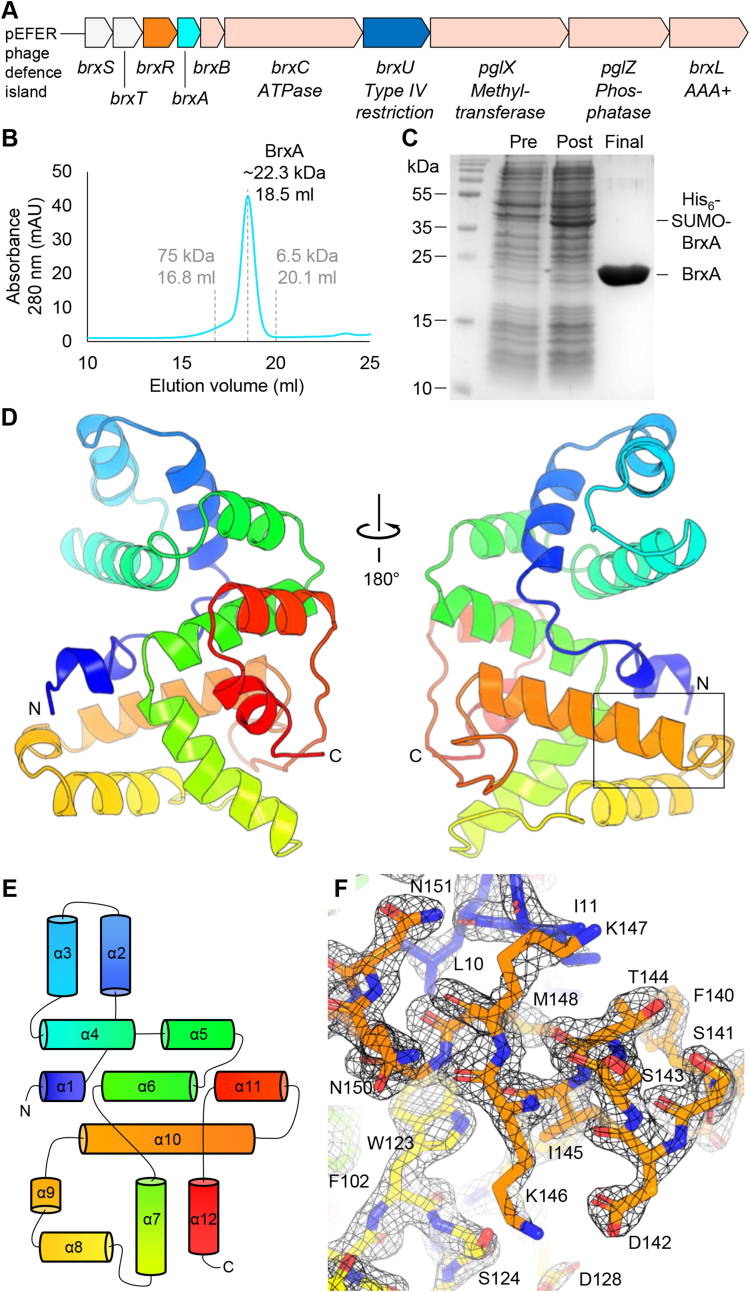
Structure of BrxA. (A) Architecture of the 17.5 ​kb phage defence island from *Escherichia fergusonii* ATCC 35469 plasmid pEFER. (B) Elution volume of untagged BrxA during analytical size-exclusion chromatography (SEC) shows it is a monomer in solution. No additional peak was observed. Calibration standards are indicated in gray. (C) SDS-PAGE of pre-induction (Pre), post-induction (Post), and cleaved, purified BrxA protein (Final). (D) Cartoon overview of the BrxA monomer, shown as a spectrum of color from blue (N-terminus) to red (C-terminus). Two views are shown, rotated by 180°. (E) Topology of the BrxA monomer. (F) Boxed region of (D), containing helix α1, α8 and α10 as sticks, shown with a 2Fo-Fc electron density map contoured to 2σ. (For interpretation of the references to color in this figure legend, the reader is referred to the Web version of this article.)

We have recently characterised the phage defence island of multidrug-resistant plasmid pEFER from the emerging pathogen *Escherichia fergusonii* ATCC 35469 (Picton et al., 2021) (Fig. 1A). This model was chosen as pEFER encoded additional genes beyond the standard type 1 BREX complement, and so had the potential to reveal the nature of more complex defence system interactions. This was indeed the case, as analysis of phage defence provided by pEFER demonstrated complementary activity between a DNA-modification dependent type IV restriction enzyme, BrxU, and a BREX system (Picton et al., 2021). These systems have been found to be co-regulated by BrxR, the archetypal member of a widespread family of WYL-domain containing transcriptional regulators (Blankenchip et al., 2022; Luyten et al., 2022; Picton et al., 2022). In this study, we make the first report of a crystal structure for a conserved core BREX protein, BrxA, found in BREX types 1, 3, 5 and 6. Downstream analyses of BrxA homolog structures identified key features of the globular fold and allowed demonstration of BrxA biological activity.

## 2 Materials and Methods

### 2.1 BrxA cloning

Total genomic DNA (gDNA) of *E. fergusonii* ATCC 35469 was obtained from ATCC. The *brxA* gene was amplified from plasmid pEFER (as part of the gDNA preparation) with primers TRB845 (5′-CAACAGCAGACGGGAGGTATGAATATAAAAGAATATTTA-3′) and TRB846 (5′-GCGAGAACCAAGGAAAGGTTATTATATTGTGCACTCCATGACCTC-3′), then cloned into pSAT1-LIC (Cai et al., 2020) via ligation-independent cloning (LIC) (Aslanidis and de Jong, 1990), to produce pTRB470. The pSAT1-LIC plasmid features a LIC site that fuses a cleavable N-terminal His_6_-SUMO tag to the target protein.

### 2.2 Recombinant protein expression

BrxA was expressed in *E. coli* ER2566 transformed with pTRB470. Overnight cultures were re-seeded 1:100 into 2 ​L baffled flasks containing 1 ​L 2 ​× ​YT. Cells were grown at 160 ​rpm, 37 ​°C, until an OD_600_ of 0.6 was reached. Expression was induced by the addition of IPTG (1 ​mM), then cells were left to grow overnight at 18 ​°C, with shaking at 160 ​rpm.

### 2.3 Recombinant protein purification

Following overnight expression, bacteria were harvested by centrifugation at 4200*g*, 4 ​°C, and the pellets were resuspended in buffer A [20 ​mM Tris-HCl (pH 7.9), 500 ​mM NaCl, 30 ​mM imidazole, and 10% glycerol]. Cells were lysed by sonication at 40 kpsi and then centrifuged at 45,000 ​*g*, 4 ​°C. The clarified lysate was then passed down a HisTrap HP column (Cytiva) using a peristaltic pump. The resin-bound protein was first washed for 10 column volumes with buffer A, followed by 10 column volumes of buffer B [20 ​mM Tris-HCl (pH 7.9), 100 ​mM NaCl, 5 ​mM imidazole, and 10% glycerol] and then eluted directly onto a HiTrap Q HP column (Cytiva) with buffer C [20 ​mM Tris-HCl (pH 7.9), 100 ​mM NaCl, 250 ​mM imidazole, and 10% glycerol]. The Q HP column was washed briefly with 5 column volumes of buffer B [20 ​mM Tris-HCl (pH 7.9), 100 ​mM NaCl, 5 ​mM imidazole, and 10% glycerol], and then transferred to an Äkta Pure (Cytiva). Proteins were separated using an elution gradient from 100% buffer B to 60% buffer D [20 ​mM Tris-HCl (pH 7.9), 1 ​M NaCl, and 10% glycerol]. Fractions corresponding to the chromatogram protein peak were pooled and incubated overnight at 4 ​°C with hSENP2 SUMO protease to cleave the N-terminal His_6_-SUMO tag from recombinant BrxA. The next day, the sample was passed through a second HisTrap HP column via a peristaltic pump, then washed for 2 column volumes with buffer B. The flow-through and wash fractions containing untagged BrxA were collected and concentrated, then loaded onto a HiPrep 16/60 Sephacryl S-200 size exclusion column (Cytiva) connected to an Äkta Pure, in buffer S [50 ​mM Tris-HCl (pH 7.9), 500 ​mM KCl, and 10% glycerol]. Fractions corresponding to the chromatogram peak were analyzed by SDS-PAGE, with optimal fractions then pooled and dialyzed overnight at 4 ​°C into buffer X [20 ​mM Tris-HCl (pH 7.9), 150 ​mM NaCl, and 2.5 ​mM dithiothreitol (DTT)] for crystallography. Crystallography samples were concentrated, quantified, and stored on ice, then either used immediately or flash-frozen in liquid N_2_ for storage at −80 ​°C. The final product was analyzed by size exclusion chromatography using a Superose™ 6 10/300 ​GL analytical size exclusion column (Cytiva) at a flow rate of 0.5 ​ml/min using buffer T [20 ​mM Tris-HCl (pH 7.9), 300 ​mM KCl].

### 2.4 Protein crystallization

Crystallization was performed using a range of commercially available screens (Molecular Dimensions). BrxA at 12 ​mg/ml was set in 200:100 ​nl and 100:100 ​nl protein:precipitant drops in MRC 2-drop 96-well plates using a Mosquito Xtal3 robot (SPT Labtech). Small rod-shaped crystals were observed in BCS screen D7 [0.2 ​M (NH_4_)_2_SO_4_, 0.1 ​M N-(2-acetamido)iminodiacetic acid (ADA, pH 6.5), 18% v/v PEG Smear High]. BrxA crystals were harvested directly from crystallization trial plates using nylon loops. Crystals were mounted into loops and then placed into a 2 ​μl drop of D7 cryo buffer [80% (v/v) BCS D7, 20% (v/v) glycerol] for 10 ​s before flash freezing in liquid nitrogen.

### 2.5 Data collection and structure determination

Diffraction data were recorded at 100 ​K on beamline I24 at Diamond Light Source. Three, 360°, datasets obtained from the same BrxA crystal were merged and processed using XDS (Kabsch, 2010), and then AIMLESS in CCP4 (Winn et al., 2011) was used to corroborate the space group. The crystal structure of BrxA was solved by molecular replacement in PHASER (McCoy et al., 2007) after generating an optimized search model using CHAINSAW (Stein, 2008) to select, conserve, and mutate residues in the 3BHW starting model according to a CLUSTALW (Larkin et al., 2007) protein sequence alignment with BrxA. Initial model-building was done using Buccaneer (Cowtan, 2006) in CCP4 (Winn et al., 2011). Data processing then moved to PHENIX (Adams et al., 2010) and COOT (Emsley and Cowtan, 2004), where the model was iteratively refined and built, respectively. The quality of the final model was assessed using COOT and the wwPDB validation server (Gore et al., 2012). Structural figures were generated using PyMol (Schrödinger). Structural superpositions were performed in PyMol via the “super” command, using full protein chains to perform a sequence-independent structure-based dynamic programming alignment followed by a series of refinement cycles to improve the fit. AlphaFold predictions were performed using default settings of AlphaFold Colab, running AlphaFold v2.1.0 (Jumper et al., 2021).

### 2.6 Electrophoretic mobility shift assays (EMSAs)

Proteins were diluted to appropriate concentrations using buffer X [20 ​mM Tris-HCl (pH 7.9), 150 ​mM NaCl, and 2.5 ​mM DTT]. Each binding reaction contained 4 ​μl of 5 ​× ​EMSA binding buffer [750 ​mM KCl, 50 ​mM Tris-HCl (pH 8.0), 2.5 ​mM EDTA (pH 8.0), 0.5% Triton X-100, 1 ​mM DTT, 55% glycerol], and 200 ​ng of phage Lambda genomic DNA (NEB). 2 ​μl of diluted protein or buffer control were added and allowed to distribute for 5 ​min on ice. Samples were diluted with water to a final reaction volume of 20 ​μl before incubation at 20 ​°C for 30 ​min. BrxA binding reactions were titrated at final protein concentrations from zero to an upper limit of 500 ​nM in 2-fold dilutions. Negative control experiments using BrxR and MenT_3_ were run at final protein concentrations of 250 ​nM, and BrxR and MenT_3_ were produced as described (Cai et al., 2020; Picton et al., 2022). Samples were loaded into a 0.7% agarose 1 x TAE gel and run at 45 ​V for 16 ​h in 1 x TAE at room temperature. The gel was subsequently post-stained in 100 ​mL 1 x TAE and ethidium bromide at a final concentration of 0.5 ​μg/mL for 30 ​min and then de-stained in 100 ​mL 1 x TAE for 30 ​min. Experiments were visualised using a BioRad ChemiDoc XRS+ ​system.

## 3 Results and discussion

### 3.1 Overall structure of BrxA

BrxA was expressed and purified as described (Materials and Methods). The final purified BrxA protein was examined by analytical size exclusion chromatography, and the elution volume corresponded closely to the predicted M_r_ of 22.7 ​kDa for BrxA, indicating the protein is a monomer in solution (Fig. 1B). This final BrxA product was also judged by SDS-PAGE to be sufficiently pure for crystallization (Fig. 1C). Using this sample, we were able to crystallize BrxA and obtained an X-ray diffraction dataset to 2.09 ​Å. The BrxA sequence was analyzed using PHYRE 2.0 (Kelley et al., 2015) to identify potential molecular replacement search models. This produced a high confidence match against PDB entry 3BHW, an uncharacterized protein from *Magnetospirillium magneticum* AMB-1 that had been solved as part of work by the New York SGX Research Center for Structural Genomics. This same entry had also previously been identified as a BrxA homolog (Goldfarb et al., 2015). Using 3BHW, we solved the structure (Fig. 1D), and refined the model to an R-factor of 0.2230 and an R-free of 0.2651 (Table 1).

**Table 1:** Data collection and refinement statistics for BrxA.

PDB ID code	7ZGE
*Data Collection*	
Beamline	Diamond I24
Wavelength (Å)	0.9795
Resolution range (Å)^a^	42.35–2.09 (2.17–2.09)
Space group	*C*2
Unit cell, *a b c* (Å); α β γ (°)	174.42, 42.54, 86.84; 90, 102.74, 90
Total reflections^a^	72060 (5602)
Unique reflections^a^	37354 (2867)
Multiplicity^a^	1.9 (2.0)
Completeness (%)^a^	100 (99.7)
Mean *I*/σ(*I*)^a^	4.4 (0.4)
*R*_*merge*_^a^^,^^b^	0.079 (0.374)
CC_1/2_^a^	0.984 (0.756)
*Refinement*	
*R*_*work*_^a^^,^^c^	0.2230 (0.3498)
*R*_*free*_^a^^,^^c^	0.2651 (0.3729)
Number of non-hydrogen atoms	4865
macromolecules	4663
ligands	0
solvent	202
Protein residues	583
RMS (bonds, Å)	0.008
RMS (angles, °)	1.12
Ramachandran favored (%)	95.64
Ramachandran allowed (%)	4.36
Ramachandran outliers (%)	0.00
Rotamer outliers (%)	0.00
Clashscore	10.16
Average B-factor	46.78
macromolecules	46.74
ligands	0.00
solvent	47.53

a Statistics for the highest resolution shell are shown in parentheses.

b *R*_merge_ ​= ​Σ_h_Σ_i_|*I*_*h*_*,*_*i*_*-I*_*h*_|/Σ_h_Σ_i_*I*_*h*_,_*i*_, where *I*_*h*_ is the mean intensity of the *i* observations of symmetry related reflections of *h*.

c R_work_/R_free_ = Σ|F_obs_-F_calc_|/ΣF_obs_, where F_calc_ is the calculated protein structure factor from the atomic model (R_free_ was calculated with 5% of the reflections selected).

There were three BrxA protomers within the asymmetric unit. As calculated using PISA (Krissinel and Henrick, 2007), contacts were minimal between each protomer, with only 409.2 ​Å^2^ and 521.5 ​Å^2^ of buried surface area between protomers A ​+ ​B, and A ​+ ​C, respectively. The Complex Formation Significance Score (scored from 0 to 1) was 0 for both interfaces (and other PISA-modelled interfaces), implying that they do not play any role in complex formation and seem to be a result of crystal packing only. Protomers B and C do not make contact within the asymmetric unit. The further PISA analysis of course does not preclude BrxA from forming oligomers if entering into complexes with other proteins or indeed nucleic acids, but does fail to identify any clear surface where oligomerization would occur. This, together with the sizing data, indicated the contacts are crystallographic and BrxA is indeed a monomer. All BrxA residues including the initial methionine (199 amino acids (aa) in total) are resolved in protomers A and B, whilst protomer C omits residues 29–36 and 47–52, inclusive. The BrxA monomer comprises a completely α-helical globular protein (Fig. 1D). BrxA is comprised of 12 α-helices; α1 (aa I3-L7), α2 (aa T18-K29), α3 (aa E33-Q43), α4 (aa G51-I65), α5 (aa D70-A78), α6 (aa E81-H95), α7 (aa 97–113), α8 (aa A122-A133), α9 (aa A135-G138), α10 (aa D142-S159), α11 (aa P177-L186), and α12 (aa E189-E196) (Fig. 1E). The helices can be considered to form bundles: α2, α3 and α4, and α8, α9 and α10 form two, 3-helical bundles, supported by a plane formed by helices α5, α6 and α7 stacking vertically through the centre of the fold. With these bundles on one face (Fig. 1D, right) the remaining helices α11 and α12 stack against the other side of α5, α6 and α7 (Fig. 1D, left). A 2Fo-Fc density map shows clear resolution of sidechains in the selected region around α1, α8 and α10 (Fig. 1F), corroborated by a composite omit map (Fig. S1).

### 3.2 Analysis of the BrxA monomer

**Fig. 2:**
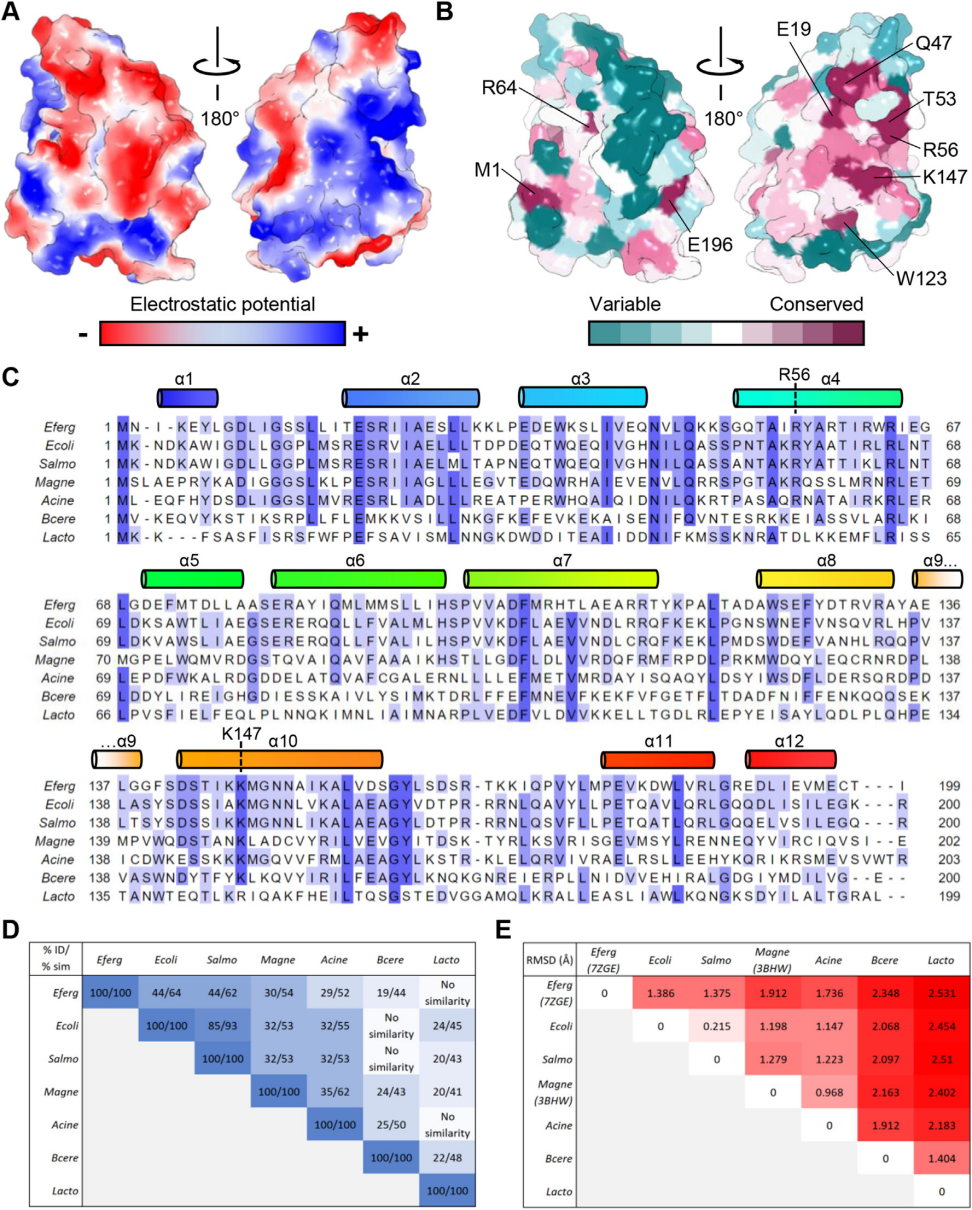
Analysis of BrxA monomers. (A) Electrostatic surface potential shows electronegativity (red) on the “front” of BrxA (left panel). There is an electropositive groove on the “back” of BrxA (right panel). (B) Conservation plots on a BrxA monomer (colored green to purple as per scale). (C) Sequence alignment of BrxA homologs, with secondary structure elements from *E. fergusonii* BrxA shown above. Shading in the alignment indicates conservation. *Eferg*, *E. fergusonii* ATCC 35469 pEFER; *Ecoli*, *E. coli* HS2; *Salmo*, *Salmonella* LT2; *Magne*, *M. magneticum* AMB-1; *Acine*, *Acinetobacter* NEB394; *Bcere*, *Bacillus cereus* H3081.97; *Lacto*, *Lactobacillus casei* Zhang. (D) Scoring matrix of BLASTp results against BrxA homologs, shown as percentage aa identity and percentage aa similarity. (E) Scoring matrix of sequence-independent superpositions for AlphaFold models of BrxA homologs, except for BrxA from *E. fergusonii* (PDB: 7ZGE, this study), and *M. magneticum* (PDB: 3BHW) where experimentally determined models were used. Values are RMSD in Å. (For interpretation of the references to color in this figure legend, the reader is referred to the Web version of this article.)

Next, we examined the surface properties of the BrxA monomer based on both electrostatic potential (Fig. 2A), and residue conservation (Fig. 2B). The “front” of the monomer is predominantly electronegative, with some patches of electropositivity (Fig. 2A, left). When rotated 180° to visualize the “back” of the BrxA monomer, there is a clear extended patch of electropositivity running through a cleft in the globular surface, with some surrounding electronegative patches (Fig. 2A, right).

ConSurf (Ashkenazy et al., 2016) was used to calculate residue conservation from multiple alignments, and the outputs were mapped onto the BrxA surface (Fig. 2B). Interestingly, conservation showed a similar distribution to the electrostatic potential, with minimal conservation within the patches of electronegativity, and greatest conservation in regions identified as electropositive (Fig. 2B). BrxA has previously been suggested to be involved in RNA-binding (Goldfarb et al., 2015), which would be supported by the observed combined distribution of charge and residue conservation. The residues identified as being most highly conserved, E19, Q47, T53, R56, W123 and K147, are clustered in the electropositive cleft (Fig. 2B). W123 and K147 can also be seen within the presented density map (Fig. 1F).

To gain a better appreciation of conservation by sequence, we performed an alignment of BrxA aa sequences from BREX systems that have been actively investigated (Fig. 2C). Specifically, BrxA from *E. fergusonii* ATCC 35469 pEFER (Picton et al., 2021, 2022), *E. coli* HS2 (Gordeeva et al., 2019; Isaev et al., 2020), *Salmonella* LT2 (Zaworski et al., 2022), *M. magneticum* AMB-1, *Acinetobacter* NEB394 (Luyten et al., 2022), *Bacillus cereus* H3081.97 (Goldfarb et al., 2015), and *Lactobacillus casei* Zhang (Hui et al., 2019, 2022). All are annotated as domain of unknown function (DUF) 1819 proteins. Though based on a smaller subset than the database-wide automated alignment performed by ConSurf, this alignment allows us to easily visualize and compare conserved residues by secondary structure (Fig. 2C). A matrix of BLASTp (Altschul et al., 1990) alignments was constructed based on these seven sequences (Fig. 2D). This shows that BrxA homologs from *E. coli* and *Salmonella* are highly related with a sequence identity of 85%, both are also closely related to the solved BrxA from *E. fergusonii* (Fig. 2D). In contrast, homologs from *M. magneticum* and *Acinetobacter* form a second group, with the two Gram-positive homologs, from *B. cereus* and *L. casei*, forming a relatively dissimilar outgroup (Fig. 2D). This is clear from the alignment, where the five Gram-negative homologs have fifteen residues completely conserved between them, and all seven examples share a further six completely conserved residues (Fig. 2C).

Next, we explored how these distinct differences in sequence conservation would manifest in predicted structures, by using AlphaFold (Jumper et al., 2021) to first produce models for all seven sequences. All seven BrxA homologs were modelled with high confidence scores (Fig. S2). Using PyMol to perform a sequence-independent structure-based superposition of the AlphaFold model for BrxA from *E. fergusonii* against the solved structure (PDB: 7ZGE, this study) produced a root mean square deviation (RMSD) of 1.016 ​Å. This indicates a good alignment between the two. Sequence-independent superposition of the AlphaFold model of the BrxA homolog from *M. magneticum* against the solved structure (PDB: 3BHW) produced an even better RMSD of 0.523 ​Å. We then compared all AlphaFold models against each other in a similar manner, except for using the two solved structures for *E. fergusonii* BrxA and *M. magneticum* BrxA in place of predicted models (Fig. 2E). The relative RMSD values worsened for the more distant homologs, but reasonable RMSD values up to a maximum 2.531 were obtained for all superpositions, including those between homologs that had no detected sequence similarity by BLASTp, for example, *E. fergusonii* BrxA and *L. casei* BrxA (Fig. 2C and E). Collectively, these data highlight clear regions of charge and sequence conservation in BrxA homologs and demonstrate that the solved globular fold is likely similar throughout this DUF1819 family.

### 3.3 Structural comparisons of BrxA

**Fig. 3:**
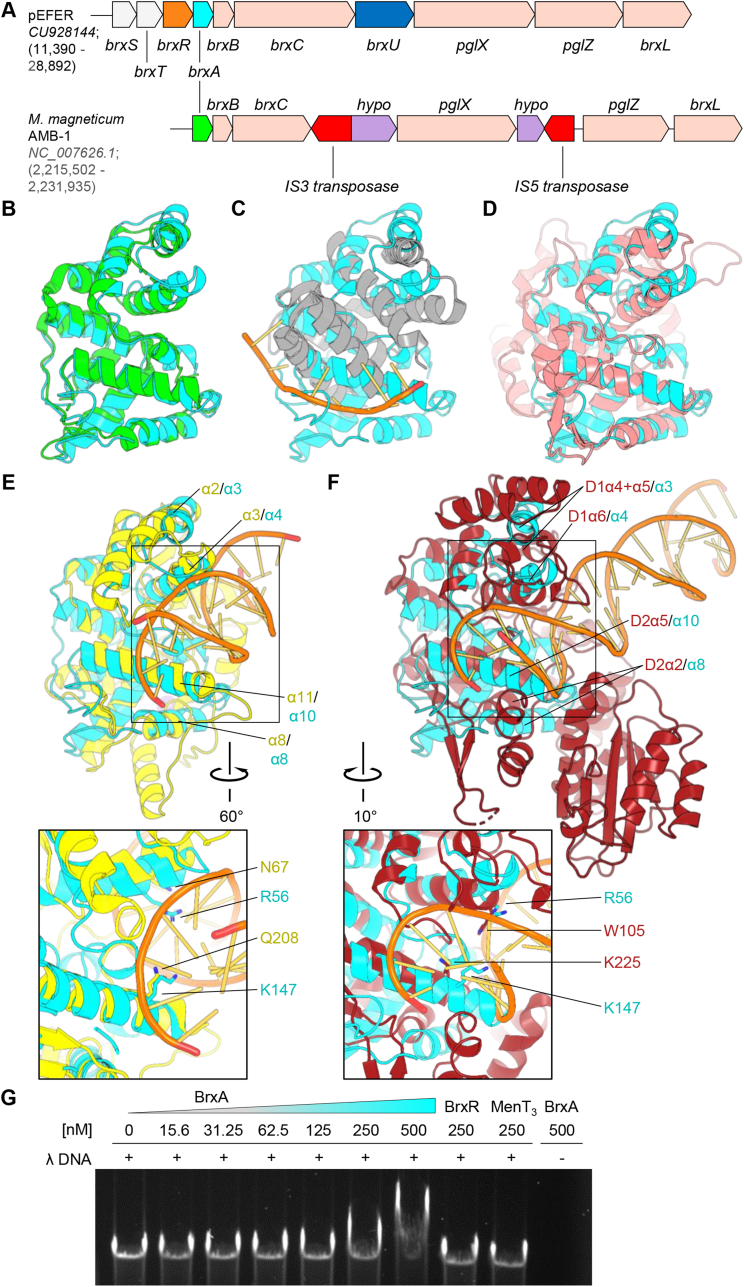
Structural homologs of BrxA. (A) Scale comparison of the 17.5 ​kb phage defence island from *Escherichia fergusonii* ATCC 35469 plasmid pEFER and the 16.4 ​kb BREX system from the chromosome of *M. magneticum* AMB-1. Genbank accession numbers and sequence positions are indicated. (B) Sequence-independent superposition of BrxA monomer (cyan, PDB: 7ZGE, this study) with BrxA from *M. magneticum* (green, PDB: 3BHW). (C) Sequence-independent superposition of BrxA monomer (cyan, PDB: 7ZGE, this study) with NusB from *Aquifex aeolicus* (gray, PDB: 3R2C). RNA bound to NusB is shown in orange. (D) Sequence-independent superposition of BrxA monomer (cyan, PDB: 7ZGE, this study) with SspB from *Streptomyces clavuligerus* (salmon pink, PDB: 6LB9). (E) Sequence-independent superposition of BrxA monomer (cyan, PDB: 7ZGE, this study) with the recognition domain of BpuJI from *Bacillus pumilis* (yellow, PDB: 2VLA). DNA bound to BpuJI is shown in orange. Inset shows a close-up of the HTH motifs. (F) Sequence-independent superposition of BrxA monomer (cyan, PDB: 7ZGE, this study) with FokI from *Planomicrobium okeanokoites* (deep red, PDB: 1FOK). DNA bound to FokI is shown in orange. Inset shows a close-up of the HTH motifs. (G) Agarose gel Electrophoretic Mobility Shift Assay (EMSA) of BrxA titrated with phage Lambda genomic DNA (200 ​ng per lane). Gel was post-stained in ethidium bromide. Protein concentration is shown above each lane. Control lanes contain either BrxR or MenT_3_ proteins, or BrxA incubated in the absence of DNA. (For interpretation of the references to color in this figure legend, the reader is referred to the Web version of this article.)

As *M. magneticum* BrxA (PDB: 3BHW), was used as a search model to solve BrxA from *E. fergusonii*, and has previously been identified as a BrxA homolog (Goldfarb et al., 2015), we wanted to examine the biological context. A scale alignment of the phage defence island from *E. fergusonii* plasmid pEFER and the chromosomal region of *M. magneticum* demonstrates that the latter encodes a type 1 BREX system that features the canonical six genes of *brxA*, *brxB*, *brxC*, *pglX*, *pglZ* and *brxL* (Fig. 3A). The defence island of pEFER is more complex than canonical BREX systems, containing an active type IV restriction enzyme that operates independently of BREX, the GmrSD-family homolog BrxU (Picton et al., 2021). Plasmid pEFER also encodes a WYL-domain containing transcriptional regulator BrxR (Picton et al., 2022), and two further upstream genes *brxS* (an IS3 transposase) and *brxT* (hypothetical), which were found to be required for BREX activity (Picton et al., 2021). *M. magneticum* appears to have a truncated *brxC* gene in comparison to pEFER *brxC*, and has two sites of insertions within the cluster, which contain two hypothetical genes, and both an IS3 and an IS5 transposase (Fig. 3A). It remains to be tested whether the *M. magneticum* system is active in phage defence. It should be noted that plasmid pEFER encodes at least nine predicted transposases, but the significance of the presence of these transposases is also not understood (Picton et al., 2021).

Previous analysis of BrxA from *M. magneticum* (PDB: 3BHW) identified the RNA-binding protein NusB from *Aquifex aeolicus* (PDB: 3R2C) as a structural homolog (Goldfarb et al., 2015). To investigate this conclusion, we first performed a sequence-independent superposition of BrxA from *E. fergusonii* (PDB: 7ZGE, this study) with BrxA from *M. magneticum* (PDB: 3BHW), producing an RMSD of 1.912 ​Å (Fig. 2E) and a clear close structural alignment (Fig. 3B). In contrast, sequence-independent superposition of NusB from *Aquifex aeolicus* (PDB: 3R2C) onto BrxA from *E. fergusonii* (PDB: 7ZGE, this study) gave a poor RMSD of 11.875 ​Å, and a clear absence of any arguable structural alignment (Fig. 3C). We conclude that NusB is not a structural homolog, and that the previous alignment is limited due to covering 44 aa (Goldfarb et al., 2015).

The DALI server (Holm and Sander, 1993) was used to search the PDB with *E. fergusonii* BrxA (PDB: 7ZGE, this study), in order to find structural homologs (Table S1). The top hit, with a Z-score of 23.1, was BrxA from *M. magneticum* (PDB: 3BHW) (Fig. 3B). NusB from *A. aeolicus* (PDB: 3R2C) was not picked up as a hit by DALI, though a NusB homolog from *Burkholderia thailandensis* (PDB: 6CKQ) was picked out as hit number 846, with a poor Z-score of only 2.3 (Table S1). After BrxA from *M. magneticum* (PDB: 3BHW), the next hit was SspB from the SspABCD-SspE phosphorothioate-dependent phage defence system (Xiong et al., 2020), with a Z-score of 8.0 for PDB entry 6LB9. Sequence-independent superposition of SspB (PDB: 6LB9) and BrxA from *E. fergusonii* (PDB: 7ZGE, this study) produced a modest RMSD of 5.031 ​Å, with a good portion of SspB (PDB: 6LB9) roughly aligned with BrxA from *E. fergusonii* (PDB: 7ZGE, this study) (Fig. 3D). SspB was crystallized in a dimeric state, with magnesium bound, and has reported activity as a nickase (Xiong et al., 2020). In comparison, BrxA is a monomer, had no metals bound, and enzymatic activity (if any) is currently unknown. Furthermore, key residues mutated at the SspB dimer interface and shown to be vital for SspB function (Xiong et al., 2020) have no structural equivalents in BrxA.

The third DALI hit, with a Z-score of 7.6, was the DNA recognition domain from the type IIS restriction enzyme BpuJI, PDB entry 2VLA (Sukackaite et al., 2008). Type IIS enzymes recognise an asymmetric DNA sequence and cleave both strands of double-stranded DNA at a fixed downstream position (Sukackaite et al., 2008). A sequence-independent superposition of BpuJI (PDB: 2VLA) against BrxA from *E. fergusonii* (PDB: 7ZGE, this study) also produced a modest RMSD of 5.460 ​Å (Fig. 3E). Nevertheless, due to the presence of DNA bound to BpuJI (PDB: 2VLA) we can make greater interpretations about potential BrxA activity. The superposition overlays helices of BrxA with recognition helices of identified helix-turn-helix (HTH) motifs within BpuJI (Fig. 3E). HTH motifs use a stabilization helix to support a second, “recognition” helix that inserts into the major groove of DNA (Beck et al., 2020; Hampton et al., 2018; Usher et al., 2021). Through comparison with BpuJI, it is now clear that the two bundles of helices identified on the “back” of BrxA (α2, α3 and α4, and α8, α9 and α10), wherein lie the conserved electropositive residues, are in fact HTH motifs juxtaposed by a rotation of 180° (Fig. 1D, right). Specifically, α2 stabilises α3 of BpuJI and the BrxA equivalents are α3 and α4, respectively. Similarly, α8 stabilises α11 of BpuJI, and the BrxA equivalents are α8 and α10 (Fig. 3E). These latter pairings differ from canonical HTH motifs due to additional secondary structural motifs in-between the binding helices. Due to the juxtaposition of these motifs, they are able to bind on either side of the DNA major groove. The distances between the two recognition helices within BpuJI (PDB: 2VLA), and BrxA from *E. fergusonii* (PDB: 7ZGE, this study) are ∼19.0 ​Å and ∼16.8 ​Å, respectively, indicating a wide enough groove in BrxA to bind either side of the DNA major groove. Mutagenesis studies in BpuJI demonstrated that mutants N67A and Q208A were no longer competent for DNA binding. Using the alignments to compare BpuJI and BrxA, it can be seen that N67 (BpuJI) is very close to R56 (BrxA), and Q208 (BpuJI) is aligned exactly with K147 (BrxA) (Fig. 3E, inset). This is noteworthy, as BrxA R56 and K147 are highly conserved residues (Fig. 2B and C).

The fourth DALI hit, with a Z-score of 7.5, was the full structure of type IIS restriction enzyme FokI, PDB entry 1FOK (Wah et al., 1997). A sequence-independent superposition of FokI (PDB: 1FOK) against BrxA from *E. fergusonii* (PDB: 7ZGE, this study) produced what could be considered a poor RMSD of 9.111 ​Å (Fig. 3F). However, despite this poor RMSD, due to the presence of DNA bound to FokI (PDB: 1FOK) it was again possible to make further conclusions regards the putative activity of BrxA. As for BpuJI, FokI contains two HTH motifs, each on independent DNA-binding domains termed D1 and D2 (Wah et al., 1997). The alignment of FokI with BrxA covers regions of both FokI domains D1 and D2, with the interface between the two splitting BrxA into two putative lobes, lobe 1 comprising helices α1-α5 and lobe 2 comprising helices α6-α12. The BrxA HTH motifs again match up and insert recognition helices into the superposed DNA major grooves (Fig. 3F). Helices D1 α4 and α5 stabilise D1 α6 of FokI, which in itself is less common, as the stabilization helix is split by a long linker. The equivalents are again α3 and α4 in BrxA. Similarly, D2 α2 stabilises D2 α5 of FokI, and the BrxA equivalents are again α8 and α10 (Fig. 3F). Conserved BrxA residues R56 and K147 are again closely superposed with residues W105 and K225 of FokI, respectively, both of which were identified as involved in FokI DNA-recognition (Wah et al., 1997). As the alignment of FokI and BrxA suggested that BrxA may be a bi-lobed protein, we aligned all three non-crystallographic protomers of BrxA in an attempt to see whether there could be any independent movement of each lobe. Structure-based superpositions between the protomers had very low RMSD values of between 0.337 and 0.398 ​Å. Examining the superpositions, it is clear that lobe 2 superposed very tightly, but there was clear movement within lobe 1, including a 3.8 ​Å movement of recognition helix α4 that carries conserved putative DNA-binding residue R56 (Fig. S3). This tentatively suggests that there could indeed be some movement within BrxA to accommodate nucleic acid interactions.

To test this hypothesis, we performed an electrophoretic mobility shift assay (EMSA) titrating BrxA against phage Lambda genomic DNA (Fig. 3G). At higher concentrations (250 and 500 ​nM BrxA), we were able to observe a shift in DNA migration, indicating binding by BrxA. We used BrxR, a DNA-binding protein with a specific binding sequence not present in Lambda genomic DNA (Picton et al., 2022), and the MenT_3_ nucleotidyltransferase (Cai et al., 2020), as negative controls for DNA interactions (Fig. 3G). BrxA tested alone produced no signal in these assays (Fig. 3G). Collectively, these data suggest that BrxA homologs are closely related to DNA-recognition domains of varied DNA-binding enzymes involved in phage defence, and that BrxA homologs are able to bind dsDNA.

## 4 Conclusion

In this study we have performed the first reported determination and analysis of a crystal structure for any of the conserved core proteins from widespread BREX phage defence systems. BrxA is monomeric in solution and has a wholly α-helical globular fold, which might be functionally split into two lobes. One face of BrxA appears relatively electronegative and non-conserved, whilst the other contains an electropositive cleft that is highly conserved. Comparison between predicted models of BrxA homologs demonstrated close similarity between systems despite varying levels of shared sequence identity. Curiously, whilst BrxA deletion mutants from the *E. coli* HS2 BREX locus were still viable for BREX-dependent methylation and phage defence (Gordeeva et al., 2019), BrxA deletion mutants from the *Acinetobacter* NEB394 strain were no longer active against phages (Luyten et al., 2022). This shows that in at least one case, though conserved, BrxA is dispensable for BREX activity. This could potentially be strain- and indeed phage-dependent. Our analyses have shown BrxA from *E. coli* and *Acinetobacter* to be close homologs and so the clear dichotomy of response to mutation remains to be explained. Obtaining deletion mutants throughout all BREX genes of associated phage defence islands, followed by testing against a diverse suite of phages such as those used against pEFER (Picton et al., 2021) will be necessary to clarify the role of BrxA within BREX defence.

Our obtained structure and analyses also appear to refute the previous conclusion that BrxA is a structural homolog of NusB, an RNA-binding protein (Goldfarb et al., 2015). Sequence-independent superpositions of DNA-recognition domains identified two HTH motifs in BrxA, suggesting that BrxA may be competent for DNA-binding (though this does not preclude RNA-binding). We hypothesised that DNA-binding could be facilitated by the two identified lobes of BrxA moving to accommodate specific DNA regions. BrxA was then confirmed to be competent for binding to dsDNA, using phage lambda genomic DNA as a binding substrate. This is the first functional evidence of biological activity for BrxA proteins. More experiments are now required to understand BrxA homolog preferences for nucleic acid length, sequence, DNA modifications, and if they can bind other forms of nucleic acids such as ssDNA, or RNA species. Whether BrxA activity is then further altered by becoming part of a larger complex of BREX proteins, and how this DNA-binding activity pertains to the BREX mechanism, also remains to be investigated. As BrxA appears to be involved in DNA-binding, and conserved in type 1, 3, 5 and 6 BREX systems, it is unclear what performs this role in other BREX systems. Type 2 BREX systems encode an additional HI helicase, but type 4 BREX systems have no other obvious additional nucleic acid-binding proteins (Goldfarb et al., 2015). The role of BrxA is therefore potentially not needed in these BREX types, which may work via a differing mechanism to type 1, 3, 5 and 6.

Identified structural homologs of BrxA are nickases (Xiong et al., 2020) or cause double-strand breaks (Sukackaite et al., 2008; Wah et al., 1997), and so BrxA should be tested for nucleic acid cleavage by performing further assays in the presence of additional metal co-factors. Finally, as BpuJI and FokI both recognise asymmetric DNA sequences, and the BREX mechanism is dependent on recognition of 6 bp non-palindromic sequences, it is tantalising to hypothesise that BrxA might in some way be involved in this recognition. This new understanding of BrxA structure suggests a conserved mode of DNA-recognition has become widespread and implemented by diverse phage defence systems. Further nucleic acid binding and cleavage studies are now required to further explore this hypothesis.

## Accession number

The crystal structure of BrxA has been deposited in the Protein Data Bank under accession number 7ZGE.

## CRediT authorship contribution statement

**Izaak N. Beck:** Investigation, Visualization, Writing – original draft. **David M. Picton:** Investigation, Visualization, Writing – original draft. **Tim R. Blower:** Conceptualization, Funding acquisition, Supervision, Investigation, Visualization, Writing – original draft.

## Declaration of competing interest

The authors declare that they have no known competing financial interests or personal relationships that could have appeared to influence the work reported in this paper.
