# Supplementary material for: Crystal structure of the BREX phage defence protein BrxA
Source: Curr Res Struct Biol. 2022 Jun 8;4:211–9. doi: 10.1016/j.crstbi.2022.06.001 (PMC9240713; doi:10.1016/j.crstbi.2022.06.001)

**Supplementary Data**

**Crystal structure of the BREX phage defence protein BrxA**

Izaak N. Beck^a,1^, David M. Picton^a,1^, Tim R. Blower^a,*^

^a^Department of Biosciences, Durham University, Stockton Road, Durham, DH1 3LE, UK

^1^These authors contributed equally to this work

^*^To whom correspondence may be addressed. Email: [timothy.blower@durham.ac.uk](mailto:timothy.blower@durham.ac.uk), tel: +44(0)1913343923.


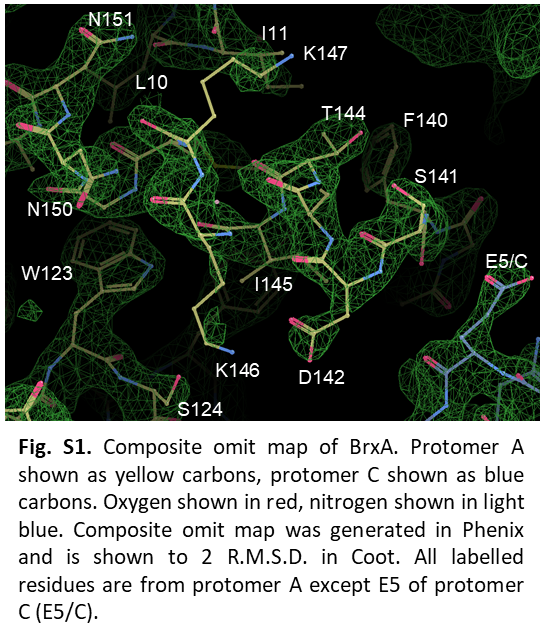


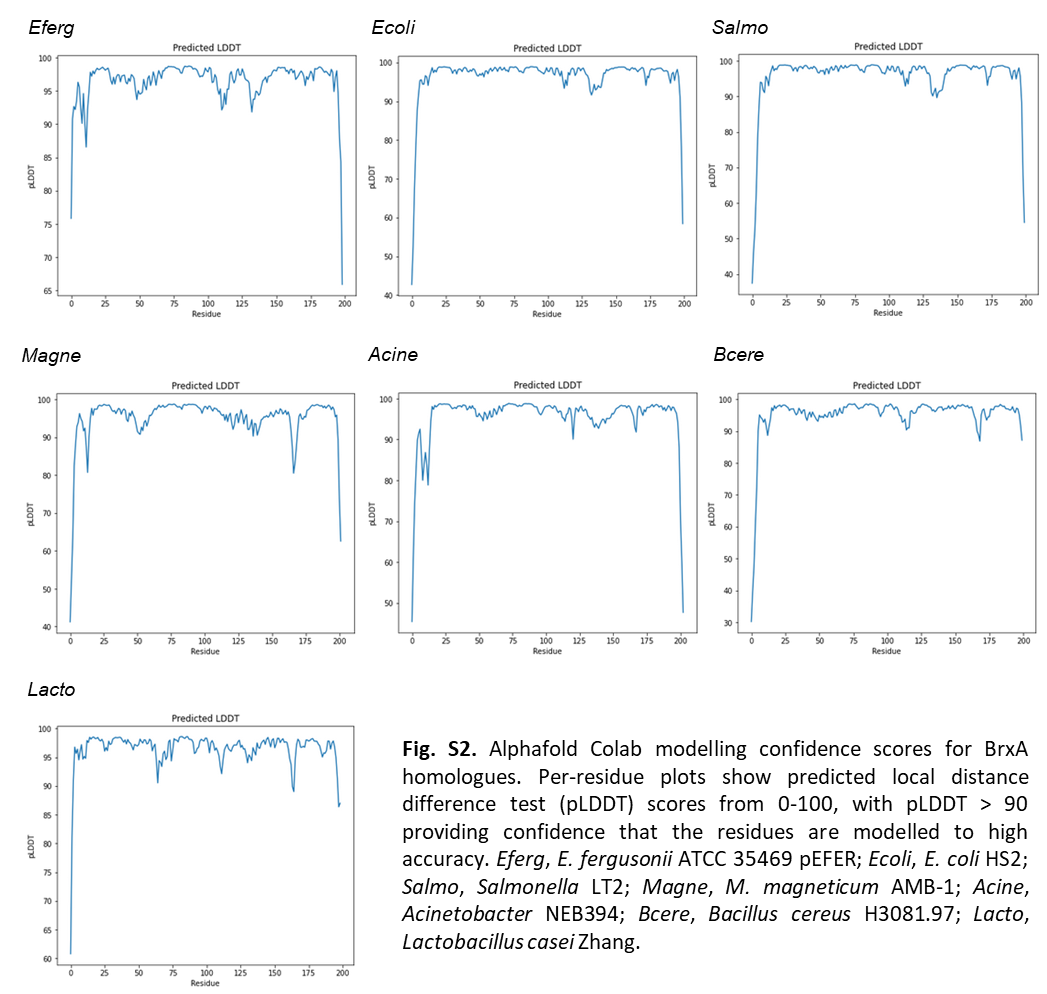


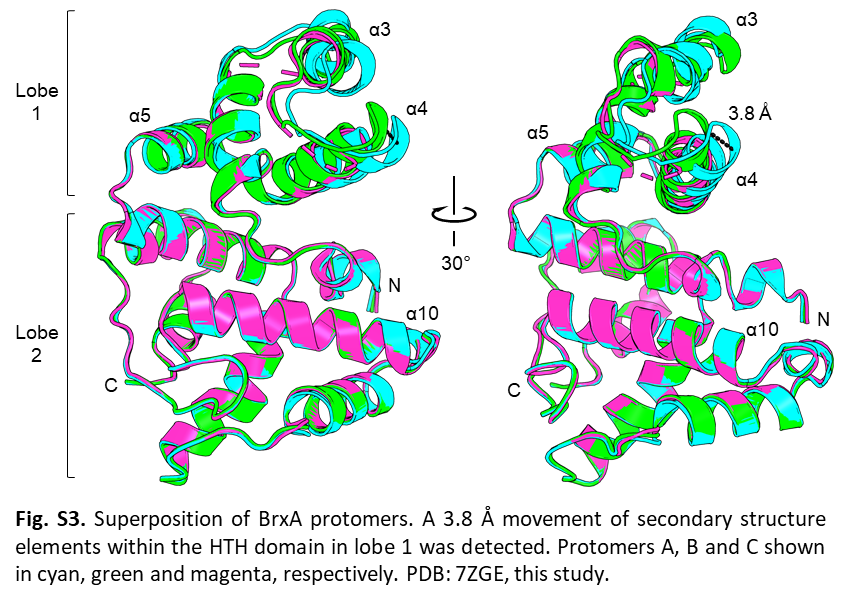

Supplement: Multimedia component 1 [file mmc1.docx]
